# Primary Pancreatic Lymphoma: Endosonography-Guided Tissue Acquisition Diagnosis

**DOI:** 10.7759/cureus.34936

**Published:** 2023-02-13

**Authors:** Anna Carolina Orsini-Arman, Rodrigo Cañada T Surjan, Filadélfio E Venco, José C Ardengh

**Affiliations:** 1 Internal Medicine, Pontifical Catholic University of Campinas, Campinas, BRA; 2 Surgery Department, University of São Paulo, São Paulo, BRA; 3 Surgery Department, Dasa/Hospital Nove de Julho, São Paulo, BRA; 4 Pathology Department, Hospital Moriah, São Paulo, BRA; 5 Gastrointestinal Endoscopy Department, Hospital das Clínicas de Ribeirão Preto, Ribeirão Preto, BRA; 6 Imaging Diagnostic Department, Universidade Federal de Sao Paulo, São Paulo, BRA; 7 Digestive Endoscopy Department, Hospital Moriah, São Paulo, BRA

**Keywords:** imunohistochemistry, diagnosis, fine needle biopsy, endoscopic ultrasound, lymphoma pancreatic

## Abstract

Primary pancreatic lymphoma is a rare type of cancer, that accounts for 0.1-0.5% of lymphomas and about 0.2% of all primary pancreatic tumors. Diffuse Large B-cell Lymphoma is the most common subtype. The diagnosis is possible if the lymphoma is located in the pancreas, but the differential diagnosis with pancreatic ductal adenocarcinoma is difficult. The diagnostic accuracy of endosonography-guided fine needle aspiration is inadequate, and thus it is common to diagnose these masses only after surgical resection. The endosonography-guided tissue acquisition allows greater accuracy in the pancreatic masses, as it determines optimal access to histological analysis using tissue in paraffin blocks for complementary immunohistochemical, and molecular tests. Thus, this elaborate diagnostic environment allows the adoption of appropriate treatment strategies for patients with this condition. The authors describe four cases of primary pancreatic lymphoma indicated for surgical resection due to suspected pancreatic cancer, with the diagnosis of Diffuse Large B-cell Lymphoma obtained by endosonography-guided tissue acquisition, changing the therapeutic strategy through the adoption of adequate chemotherapy treatment with good progress.

## Introduction

Primary pancreatic lymphoma (PPL) is a rare type of cancer, that accounts for 0.1-0.5% of lymphomas and about 0.2% of all primary pancreatic tumors (PPT) [1,2]. The non-Hodgkin ´s Lymphoma (nHL) is a heterogeneous group of lymphoproliferative disorders, that rarely involve the pancreas [3]. The overlap of both primary and secondary tumors with other types of pancreatic neoplasia occurs due to its rarity, besides its clinical and radiological characteristics, making the diagnosis difficult and preventing adequate treatment of this disease [4,5]. These tumors are frequently located in the head of the pancreas in elderly men, with Diffuse Large B-cell Lymphoma (DLBL) being the most commonly detected subtype [4,5]. The diagnosis of a PPL is possible if the lymphoma is located in the pancreas, even if there is the involvement of peripheral lymph nodes (LN) or distant dissemination. However, the diagnosis is difficult when a solid pancreatic mass, mimics a pancreatic ductal adenocarcinoma (PDA) [6-8].

The accuracy of the biopsy obtained by endosonography-guided fine needle aspiration (EUS-FNA) in PPL is inadequate, requiring several punctures, thus increasing the chance of adverse events [9]. Therefore, the diagnosis of these tumors, most of which are solid, is common only after surgical resection [10,11]. The endosonography-guided tissue acquisition (EUS-TA) appears to be an effective tool for diagnosing this neoplasia, allowing tissue samples to be obtained and consequently improving pathological anatomy results. This technique emerged after the development of thicker and more flexible needles, which assist in obtaining elongated MicroCore (MC) in larger quantities, in addition to reducing the number of punctures on the mass, thus reducing the rate of adverse events. Obtaining more tissue fragments allows adequate histological examination with routine staining, along with the use of auxiliary methods such as immunohistochemistry, genetic and molecular tests. For flow cytometry (FC), the collected material is sent fresh in an appropriate transport liquid [12]. Precise diagnosis helps in early chemotherapy treatment, improving the prognosis of the disease [4,5].

We report four cases of DLBL indicated for surgery due to suspected imaging exams of PDA and accurately diagnosed by EUS-TA, allowing chemotherapy treatment with good evolution.

## Case presentation

Case 1

**Figure 1:**
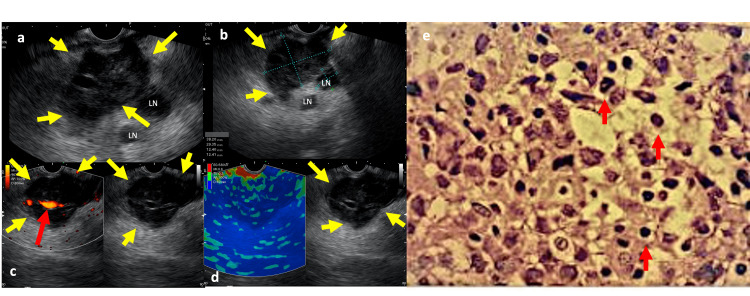
Endosonographic and histology images (a) Hypoechoic, heterogeneous solid nodule with imprecise borders (yellow arrows) and peripheral lymph nodes. (b) Node (yellow arrows) measuring 38 x 29 mm and lymph nodes of 12 x 12 mm. (c) Doppler (+) of vascular structure encompassed by the mass (red arrow). (d) Elastography showing a stiff pancreatic nodule. (e) Histology – microscopic imaging of Diffuse Large B-cells Lymphoma (red arrow).

A 59-year-old man with a history of hypertension was admitted to the hospital for umbilical herniorrhaphy. In the preoperative evaluation, the patient was asymptomatic. Physical examination didn’t identify palpable adenopathy and the abdomen was flaccid, painless, and without visceromegaly. The abdominal ultrasonography (US) showed a hypoechoic and irregular perisplenic nodule measuring 42 mm. The abdominal MRI identified a poorly delimited pancreatic infiltrative lesion involving the splenic artery, which extended to the pancreatic tail, measuring 39 x 29 mm, associated with multiple splenic lesions of up to 40 mm. Also, the exam showed an enlarged spleen with infarction areas and retroperitoneal lymph node enlargement on the left, both peri-aortic and intercavoaortic (33 mm). Laboratory tests were normal, including CA 19.9 = 36 U/mL (normal value {nl} = 37 U/mL), lactate dehydrogenase (LDH) = 178.3 U/L (nl= 125.00 U/L e 220.00 U/L), and carcinoembryonic antigen (CEA) = 1.8 ng/mL (nl = 3.5 ng/mL). The endoscopic ultrasound (EUS) showed a hypoechoic and heterogeneous area in the tail of the pancreas, Doppler (+), measuring 38 x 29 mm, with involvement of vascular structures. Elastography showed stiffness of the lesion. The area extended to the celiac trunk and showed a 12 mm perilesional lymph node enlargement (Figure 1). The tissue acquisition (TA) was performed with a ProCore 20G needle (EchoTip® Ultra Endoscopic Ultrasound Needle Cook Medical, Bloomington, IN, USA), obtaining a large amount of material. The immunohistochemical panel showed DLBL. Positron emission tomography-computed tomography (PET-CT) revealed anomalous fluorodeoxyglucose F 18 (18F-FDG) concentration in the right tracheobronchial and hilar lymphadenopathy, located in an area of lymph node conglomerate compromising the tail of the pancreas with periaortic celiac, mesenteric and splenic block, as well as left para-aortic adenopathy. The patient started six cycles of chemotherapy treatment (R-CHOP {rituximab, cyclophosphamide, doxorubicin, vincristine, and prednisone}). There was complete remission of the disease. The patient is still under outpatient follow-up (36 months) and his disease is under control.

Case 2

**Figure 2:**
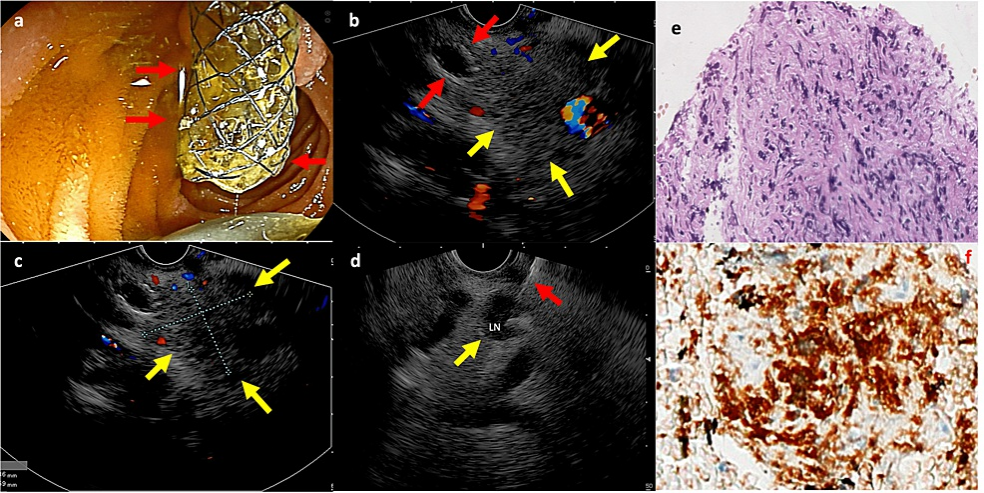
Endoscopic and immunohistochemistry examination images (a) Endoscopic Ultrasound equipment in front of the duodenal papilla, with the metallic stent exiting the duodenal papilla (red arrows). (b and c) Endoscopic Ultrasound imaging:  metallic stent inside the common bile duct (red arrows); hypoechoic and heterogeneous area (34 x 22 mm) of imprecise limits (yellow arrows) and Doppler (+). (d) Lymph node measuring 12 x 11 mm (yellow arrow), with the puncture needle (red arrow) inside. (e) Diffuse Large B-cell Lymphoma (Hematoxilin and Eosin – 200X) e (f) Immunohistochemistry with CD 20 (+).

A 79-year-old man was admitted with abdominal pain, jaundice, lack of appetite, and a weight loss of 6 kg in four months. In his first appointment, laboratory tests showed total bilirubin of 6.4 mg/dL, with direct bilirubin of 4.8 mg/dL, alkaline phosphatase of 198.4 U/L, aspartate aminotransferase (AST) of 345 U/L, alanine aminotransferase (ALT) of 278 U/L, LDH = 234 U/L (nl= 125.00 U/L e 220.00 U/L), leukocytosis with a left shift and a fever of 38.1 ºC. The abdominal MRI/MRCP (magnetic resonance cholangiopancreatography) revealed a mass in the head of the pancreas with poorly defined borders and dilation of the common bile duct, associated with peripancreatic lymph node enlargement. Due to the diagnosis of cholangitis, endoscopy was indicated for drainage of the biliary tract. The endoscopic retrograde cholangiopancreatography (ERCP) showed intrapancreatic bile duct stenosis and after papillotomy it was possible to implant a fully covered self-expandable metallic stent (SEMS). Immediately after endoscopic drainage, EUS-TA was performed. The procedure revealed a heterogeneous area in the cephalic portion with imprecise limits, measuring 34 x 22 mm and in close contact with the superior mesenteric vein without invading it (Figure 2). EUS-TA was performed with a 20G ProCore needle (EchoTip® Ultra Endoscopic Ultrasound Needle Cook Medical, Bloomington, IN, USA) and several MCs were obtained. The specimen's immunohistochemical profile revealed DLBL with Ki-67 (+) at > 80 % and CD20 (+) (Figures 2e, 2f). The PET-CT showed anomalous concentration of 18F-FDG in the mass located in the head of the pancreas and in the hepatic hilum. The patient started R-CHOP (six cycles) of chemotherapy treatment. There was remission of the disease and the follow-up (34 months) showed that her disease is under control.

Case 3

**Figure 3:**
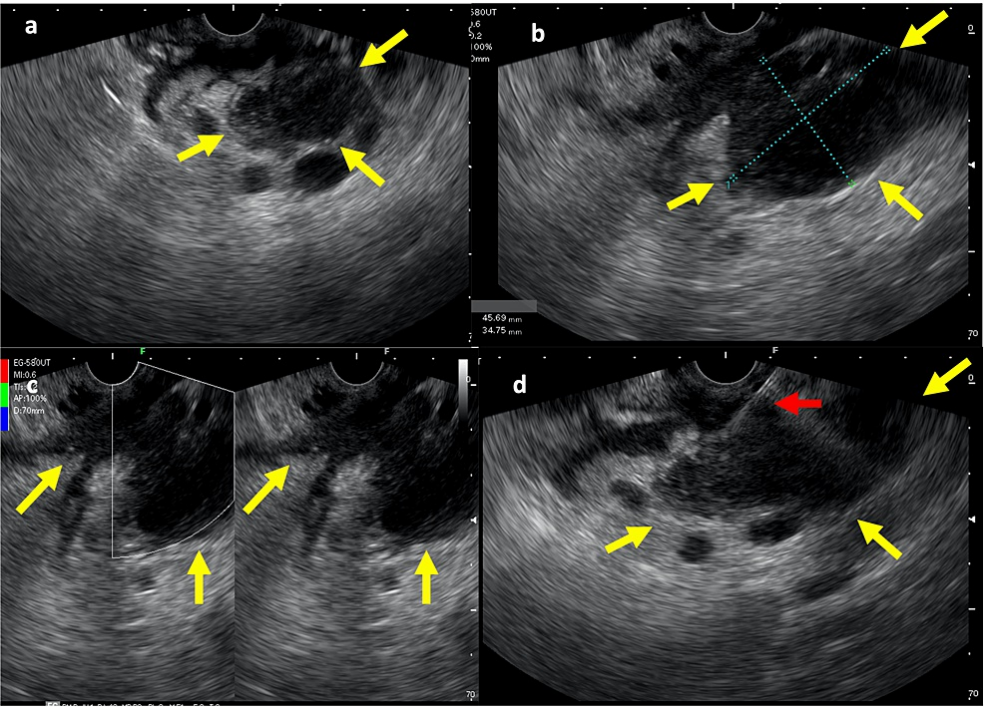
Endoscopic ultrasound imaging (a) hypoechoic and heterogeneous area with poorly defined limits (yellow arrows). (b) solid-cystic, irregular lesion located at the ill-defined body/tail transition, measuring 45 x 34 mm (yellow arrows). (c) Solid-cystic lesion without Doppler signal affecting the splenic artery (yellow arrows). (d) Insertion of the puncture needle for tissue acquisition (red arrow), within the limits of the lesion (yellow arrow).

**Figure 4:**
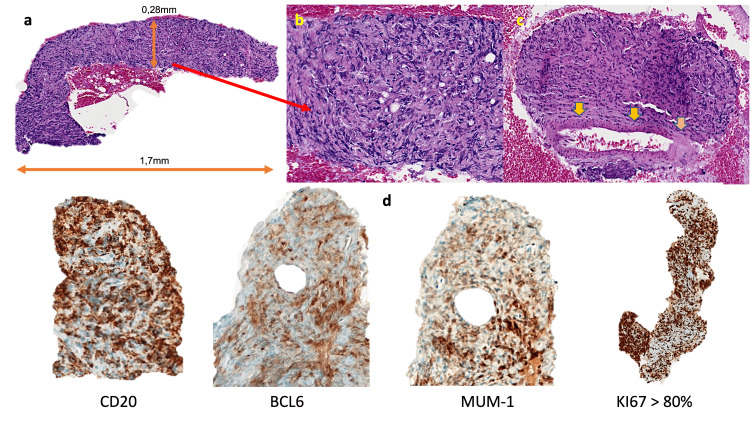
Immunohistochemistry examination images (a) Tissue acquisition obtained during Endoscopic Ultrasound with the ProCore 20G needle. The specimen is 0.28 mm wide and 1.7 mm long. (b) Hematoxylin and Eosin – 200x magnification with large amounts of material. (c) Lymphoma with areas of sclerosis and vascular invasion (yellow arrows). (d) The Immunohistochemical profile determines the histogenesis of the lymphoma and subclassifies it into Diffuse Large B-cell Lymphoma of non-germinal center origin (see Table 2)

A 71-year-old woman came to the hospital due to abdominal pain radiating to the back. She underwent clinical treatment but didn’t show improvement. Complementary CT and MRI revealed the presence of a mass in the body of the pancreas measuring approximately 45 x 34 mm. Laboratory tests were normal, including LDH = 136.1 U/L (nl= 125.00 U/L e 220.00 U/L), Ca 19.9 = 27 U/mL (nl = 37 U/mL) and CEA = 0.8 ng/mL (nl = 3.5 ng/mL). EUS was indicated in this case, which showed a poorly delimited solid-cystic lesion in the body/tail transition region with adjacent anechoic areas, Doppler (-), measuring 45 x 34 mm and affecting the splenic artery. Elastography showed stiffness of the lesion (Figure 3). The EUS-TA was performed with a 20G ProCore needle, obtaining a large amount of material from the solid areas and about 10 cc of serohematic fluid, which was sent to biochemical analysis (CA19.9 = 12,000 U/mL, CEA = 198 ng/mL, amylase = 278 U/dL and glucose = 107 mg/dL) of the aspirated fluid. The immunohistochemistry (IHC) profile determines the histogenesis of the lymphoma and subclassifies it into DLBL of non-germinal center origin (Figure 4) [13]. The patient started chemotherapy treatment. The patient experienced adverse effects and was able to complete four cycles of the R-CHOP protocol. Outpatient controls showed partial remission of the disease with a 17 month follow-up.

Case 4

**Figure 5:**
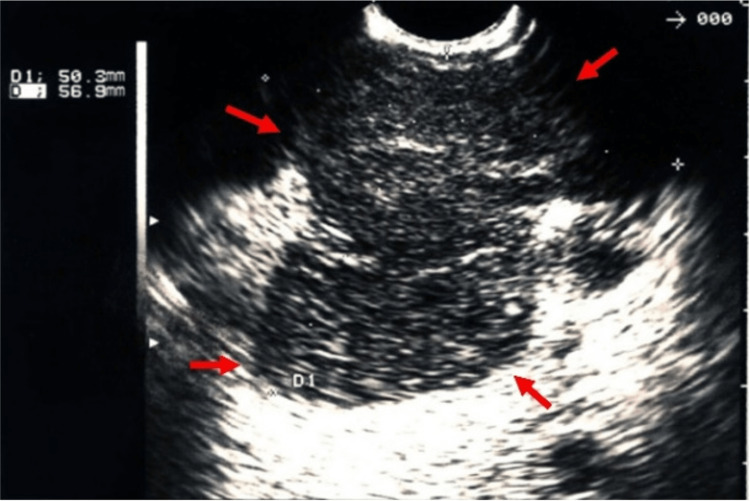
Endoscopic ultrasound imaging The image shows a solid-cystic lesion (red arrows), measuring 56 x 50 mm in the largest axes, located in the body of the pancreas.

A 32-year-old woman was admitted to the hospital with fatigue and an afternoon fever for the last 15 days. Physical examination revealed a distended abdomen and pain on superficial palpation. The US revealed a large amount of air inside the loop, which made the examination of the retroperitoneum difficult. Also, the CT scan revealed the presence of a solid area in the region of the body towards the tail measuring about 38 x 32 mm. These findings were confirmed by MRI which displayed a mass in the body of the pancreas measuring approximately 36 x 35 mm. Tumor markers were requested and showed LDH = 321 U/L (nl= 125.00 U/L e 220.00 U/L), CA 19.9 = 41 U/mL (nl to 37 U/mL) and CEA = 0.5 ng/mL (nl = 3.5 ng/mL). The EUS was also indicated for this patient, which showed a solid-cystic, poorly delimited, bilobed lesion in the body/tail transition region, measuring 41 x 35 mm with adjacent anechoic areas, Doppler negative, and no vascular involvement. Elastography revealed the stiffness of the mass. The EUS-TA was performed with the ProCore 20G needle, obtaining a large amount of MC from the solid areas, about 3 mL of citrine yellow liquid, which was sent for biochemical analysis revealing a CA 19.9 = 8,000 U/mL, CEA = 87 ng/mL, amylase = 167 U/dL and glucose = 89 mg/dL. The immunohistochemical panel showed high-grade nHL (Figure 5). The patient was able to complete six cycles of chemotherapy treatment using the R-CHOP protocol. However, the remission of the disease was partial. This patient was followed up for two years and then was lost to follow-up.

Table 1 displays the epidemiological characteristics of the patients, the results of CA 19.9, CEA, and the findings of imaging exams. The IHC panel performed on the four patients and the results that confirmed the diagnoses are exhibited in Table 2.

**Table 1:** Patients' characteristics and imaging methods results AP: abdominal pain; c-PAD: cystic-pancreatic adenocarcinoma; F: female; f: fever; G: gender; HE: Haematoxilin-Eosin; J: jaundice; LN: lymph node; M: male; NA: non-available; PAD: pancreatic adenocarcinoma; PM: pancreatic mass; T: tiredness; WL: weight loss.

Case	Age	G	Symptons	CA 19.9 (U/mL)	CEA (ng/mL)	CT	MRI	EUS	Type of Lesion	Site	Size (mm)
1	59	M	No	36	1.8	PM+LN	PM+LN	PAD	SOLID	TAIL	50
2	79	M	AP+J+WL	NA	0.6	PM+LN	PM+LN	PAD	SOLID	HEAD	38
3	71	F	AP	27	0.8	PM	PM	c-PAD	SOLID-CYST	BODY	45
4	32	F	T+F	41	0.5	PM+LN	PM+LN	c-PAD	SOLID-CYST	BODY	41

**Table 2:** Immunohistochemistry profile (-) negative; (+) positive; AE1/AE3: cytokeratin ae1/ae3 antibodies AGC: anaplastic giant cells; BCL6: B-cell lymphoma 6 c-kit: proto-oncogene c-kit; CD10: CD10 antigen; CD20: B lymphocyte antigen CD20; CD23: cell surface antigen; CD3: cluster of differentiation 3; CD30: tumor necrosis factor; CD34: CD34 gene; EUS-TA: endosonography-guided tissue acquisition; HE: hematoxilin & eosin; Ki-67: marker of proliferation-67; MN: malignant neoplasia; MUM1: multiple myeloma 1; NA: non-available; Pax5: B-cell specific activator protein.

CASE	HE	C-KIT	CD34	KI-67	CD20	AE1/AE3	CD3	CD10	BCL6	MUM-1	PAX-5	CD23	CD30
1	MN	(-)	(-)	(+ - 80%)	(+) AGC	(-)	(-)	NA	NA	NA	NA	NA	NA
2	MN	(-)	(-)	(+ - 80%)	(+) AGC	(-)	(+) Focal	NA	NA	NA	NA	NA	NA
3	MN	(-)	(-)	(+ - 80%)	(++) Focal	(-)	(+) Focal	(-)	(+) Focal	(+) Focal	(-)	(-)	(-)
4	MN	(-)	(-)	(+ - 80%)	(+) AGC	(-)	(-)	NA	NA	NA	NA	NA	NA

## Discussion

PPL is a rare subtype of primary malignancy, consisting of <0.5% of all pancreatic cancers, usually found in men aged 35 to 75 years [3,14]. Some diagnostic criteria are needed for an adequate classification of the PPL. First, the absence of superficial lymphadenopathy or enlargement of mediastinal lymph nodes on chest imaging is required, a fact confirmed in the four patients. Also, the leukocyte count must be normal in the peripheral blood, a fact confirmed in cases 1, 3, and 4. Although in case 2 there was an increase in the leukocyte count due to cholangitis, which affected the patient, it returned to normal three days after endoscopic drainage. Third, the main mass must be located in the pancreatic gland and the lymph node involvement must be confined to the peripancreatic region, which occurred in the four patients and lastly, the absence of hepatic or splenic involvement, which occurred in all of the patients in this series [1,2].

PPL is usually associated with nonspecific symptoms, such as jaundice, abdominal pain, and abdominal mass, rather than presenting symptoms like fever and night sweats, typical of DLBL [3,14]. Another important fact is that serum tumor markers are not useful in PPL, as they are not consistently increased, which can be observed in these patients who had normal CA 19.9. Regarding imaging exams, their findings mimic other types of pancreatic neoplasms, making the diagnosis difficult and collaborating for the delay in treatment [4,5]. In the four reported patients, all had lesions with similar characteristics to PDA on CT, MRI/MRCP, and EUS. All had indications for surgical treatment, as CT and MRI confirmed the presence and involvement of lymph nodes, pointing to a PDA diagnosis. This idea was only put aside after the diagnosis of DLBL.

The EUS-TA seems to improve diagnostic precision, with one or two passes of the needle through the tumor mass. In addition to being less invasive, it allows for a more accurate characterization of the lesion [15]. In addition, obtaining a reasonable amount of material allows the completion of other auxiliary methods such as IHC and FC, which helped in the diagnosis of this disease in the four reported cases.

As demonstrated in these cases, the histological samples of pancreatic nodes and peripancreatic lymph nodes were adequate. Consequently, it enables the diagnosis and the appropriate treatment of a PPL instead of performing surgery, which would be the correct treatment for a PDA. The good amount of collected tissue allowed the study of the tumor’s immunohistochemical panel and was also used for the FC, helping in the diagnosis [16,17]. In this regard, literature is scarce, especially related to the role of EUS-TA in the diagnosis of PPL. The authors describe the first four reported cases of DLBL using EUS-TA with the ProCore 20G needle.

This diagnosis granted the recommended first-line chemotherapy protocol R-CHOP to be implemented. It consists of the monoclonal antibody rituximab, associated with cyclophosphamide, doxorubicin, vincristine, and prednisone, exhibiting a disease-free survival rate of 50 to 80% [18,19]. The authors report four patients with PPL due to nHL of the DLBL type, which is an aggressive lymphomatous disease that rapidly leads to the deterioration of the patient’s clinical condition. All lesions were detected by EUS-TA before being submitted to a surgical procedure. Among the described cases, an unfavorable factor was the unusual involvement of the pancreas, which is commonly related to delayed diagnosis and worsened prognosis [16,17]. The treatment of the four presented cases of PPL consisted of chemotherapy cycles under the guidance of a hematologist, without the need for surgical resection [5,15,17,19]. The prognosis of PPL is better than that of PDA, which can be seen in 835 patients where the median survival was 53 (37 to 73) months, unlike the PDA cases who underwent surgery, which had an overall survival of around 24.5 months for asymptomatic patients and 11 months for symptomatic patients [20].

## Conclusions

We conclude that EUS-TA is the least invasive tool with highly effective diagnostic ability. Therefore, it should be considered when the patient presents pancreatic mass with peripancreatic lymph nodes, so that it may be possible to obtain a viable amount of tissue, thus improving the result of the anatomopathological tests. Precise histological examination is essential to distinguish a PPL from other pancreatic masses, since the treatment of PPL is conservative and based on chemotherapy, without the need for surgical resection.
